# Demographic, Clinical, and Quality of Life Profiles of Older People With Diabetes During the COVID-19 Pandemic: Cross-Sectional Study

**DOI:** 10.2196/49817

**Published:** 2023-11-16

**Authors:** Fabianne Sousa, Lucianne Nascimento de Araujo, Tainá Sayuri Onuma de Oliveira, Mateus Cunha Gomes, Glenda Ferreira, Cintia Aben-Athar, Silvio Eder Dias da Silva, Aline MP Cruz Ramos, Diego Pereira Rodrigues

**Affiliations:** 1 Nursing School Federal University of Para Belém Brazil

**Keywords:** aged, diabetes mellitus, COVID-19, cross-sectional study, quality of life, long COVID-19, diabetic, older people

## Abstract

**Background:**

Diabetes mellitus, one of the main diseases that affects the Brazilian population older than 60 years, is defined as a divergent group of metabolic disorders that present a high level of glycemia (hyperglycemia), causing damage to various organs and systems of the body, including the heart, kidneys, eyes, and nervous system. It is believed that in 2025, in Brazil alone, there will be more than 18.5 million individuals diagnosed with diabetes mellitus. Therefore, it is important to know the individuals’ quality of life in the context of life and culture.

**Objective:**

This study aimed to assess the demographic, clinical, and quality of life profiles of older adults with diabetes during the COVID-19 pandemic in a university hospital complex in the northern Amazon region.

**Methods:**

We conducted a cross-sectional, exploratory, noninterventional, descriptive, and analytical study using a nonrandom sample of 54 older people diagnosed with diabetes mellitus at the geriatrics outpatient clinic of the medium and high complexity university hospital in the western Brazilian Amazon between 2020 and 2022. We used 3 instruments, namely, a sociodemographic questionnaire, a clinical conditions questionnaire, and Diabetes-39. Qualitative data were described using absolute and relative frequencies. The Kolmogorov-Smirnov normality test was applied, and the *z* test was used for inferential analysis. SPSS software (version 27) was used for data analysis, and the significance level was 5%.

**Results:**

Of the 54 interviewees, the majority were women, married, retired, and had a good quality of life. Of these, 48.1% (n=26) were infected by COVID-19, 61.5% (n=16) of whom progressed to long COVID, presenting with fatigue or muscle weakness. As for the quality of life, the “social overload” (*P*<.001) and “sexual functioning” (*P*<.001) dimensions had with low scores compared to the “energy and mobility” (*P*=.005), “diabetes control” (*P*<.001), and “anxiety and worry” (*P*<.001) dimensions. Quality of life was negatively impacted in the “anxiety and worry” dimension. Among those affected by COVID-19, most progressed to long COVID; however, there was a lack of data on this theme in the population of older people with diabetes.

**Conclusions:**

The majority of interviewees progressed to long COVID, with their quality of life negatively impacted in the “anxiety and worry” dimension, reflecting that health actions prioritizing mental health should be implemented by health professionals.

## Introduction

The world continues to suffer a dramatic situation of catastrophic proportions due to the worldwide spread of COVID-19 caused by SARS-CoV-2 [1]. SARS-CoV-2 appears to impact people with comorbidities, for example diabetes mellitus (DM), and symptoms may persist for more than 12 weeks post–COVID-19. Among these symptoms, the most prevalent are fatigue, dyspnea, weakness, and headache; this conglomeration of persistent symptoms is called “long COVID” or “post–COVID-19 Syndrome” [2]. Studies in the international literature point out that the risk of long COVID is probably higher in women, older people, people with obesity, and patients with diabetes [2,3].

DM is a chronic disease with devastating multisystemic complications and is estimated to have inflicted 463 million people worldwide in 2019, with a projected trend of reaching 578 million people by 2030. It is estimated that 80% of people with DM live in low- and middle-income countries. Data from the Brazilian Diabetes Society indicate that there were more than 14 million people with the disease in 2015 in Brazil [4], which ranked fourth most in the world. In 2019, it was estimated that there would be 16.8 million Brazilians with diabetes between 20 and 79 years old, with a projected increase of 55% by 2045 [5,6].

In this pandemic context, the older population is a high-risk group for infection with SARS-CoV-2 as their immune systems suffer from the aging process, called immunosenescence, in which there is a reduction in the ability to respond to infections, promoting an increase in the severity of infectious diseases [7], especially in those with comorbidities. Thus, significant impacts of COVID-19 have been observed pertaining to the health and quality of life of the older population. There are several definitions of quality of life; however, the most widely used is that by the World Health Organization (WHO), which defines it as an individual’s perception of his or her position in life in the context of the culture and value system in which he or she lives and in relation to his or her goals, expectations, standards, and concerns [8]. In this sense, it becomes necessary to review public policies and strategies to ensure the quality of life of older people living with diabetes after contracting COVID-19 and strengthen health actions effectively to improve the health behaviors of individuals with diabetes [9] so that there is an efficient recovery of those affected by long COVID. Thus, the guiding question of this study was the following: what are the sociodemographic, clinical, and quality of life profiles of older people with diabetes treated at a university hospital during the pandemic? This study aimed to answer this question in a university hospital complex in the northern Amazon region.

## Methods

### Study Design

We conducted a cross-sectional, exploratory, noninterventional, descriptive, and analytical study. In addition, the STROBE (Strengthening the Reporting of Observational Studies in Epidemiology) checklist [10] for observational studies was used to help conduct the research and report the results obtained.

### Study Population and Sampling

Data collection took place in the geriatric outpatient clinic, where older people with diabetes are cared for by the geriatric medical clinic team made up of a multidisciplinary team of doctors, nurses, dentists, psychologists, and social workers. The study was conducted at a medium and high complexity hospital, the Hospital Universitário João de Barros Barreto, in the western Brazilian Amazon (city of Belem, Pará, Brazil) from September 2020 to February 2022. We considered Decree no. 10.308, of March 20, 2020, which provides for the request for goods and services provided by public sector companies linked to the Ministry of Infrastructure during the period of the state of public calamity due to the pandemic [11].

To obtain the population sample, the intentional and convenience nonrandom sampling technique was used. Therefore, doctors from the geriatrics service who carried out consultations with patients with diabetes were asked to identify all older people diagnosed with DM to carry out the interviews, considering the restrictions of face-to-face care in the hospital complex during the pandemic period.

The study inclusion criteria included the following: older people with controlled DM, those with periodic medical consultations from the DM Control Program of the Ministry of Health, those aged 60 years or older, and those with the cognitive skills to understand and answer all the questions on the instruments. The following patients were excluded: older people with diabetes with altered blood glucose control or those undergoing irregular treatment without medical supervision, hospitalized or surgical patients, those in palliative care, those whose language was different from Brazilian Portuguese, and those who were unable to respond to the questionnaires. The final sample size of the study was 54 older patients with diabetes.

Before starting the study, prior contact was made with hospital leaders to present the research and access the geriatrics outpatient clinic. The interviews were carried out in an office made available for the study that lasted an average of 30 to 40 minutes.

### Study Instruments

We applied the following 3 data collection instruments: a structured questionnaire addressing sociodemographic variables (ie, gender, age group, marital status, education, and occupation), a questionnaire comprising questions related to clinical variables regarding DM (ie, time of diabetes treatment and use of medication) and symptomatology (COVID-19 and long COVID), and the Brazilian version of the Diabetes-39 questionnaire (D-39) to assess the quality of life of patients with diabetes [12].

### Diabetes-39 Questionnaire

The D-39 is a multidimensional scale, developed in the United States, composed of 39 items that assess the health-related quality of life in relation to the following 5 domains of life pertaining to people with diabetes: energy and mobility (15 items), diabetes control (12 items), anxiety and concern (4 items), social burden (5 items), and sexual functioning (3 items). Each item is calculated from the assessment made by the person with DM, helping to identify assistance needs and, consequently, reducing the risk of complications from the disease. The scores obtained in this questionnaire followed its original version, presented as a continuous horizontal line with vertical marks that delimit the spaces where the numbers 1 to 7 are located. The scale ranged from “1 for not affected at all” and “7 for extremely affected”. Since it is a Likert-type scale of up to 7 points, it is necessary to consider the range of distribution of the answers; thus, based on previous research on the D-39 version adapted for Brazil [13,14], for our analysis, we defined the scores for quality of life as “not affected” (1 to 3 points) and “extremely affected” (4 to 7 points). The reliability of the instrument was assessed by Cronbach α, which ranged from .81 to .93 for the 5 dimensions; values greater than or equal to .70 were considered acceptable [14]. To control the quality of data collection and typing, measures were adopted, such as the training of interviewers, checking of questionnaires, and double typing.

### Ethical Considerations

The study followed the standards of the 2013 Declaration of Helsinki, ensuring confidentiality and anonymity of the data of all participants, with a favorable opinion by the Ethics Committee on Human Research of the Federal University of Pará in accordance with Resolution no. 466/2012 of the National Health Council (approval no. 4,389,533). All the patients who agreed to participate in the study were presented with the objectives, risks, and benefits of the study, and after the information was provided, they signed the Free and Informed Consent Form, received a copy of it, and proceeded to the interview.

### Statistical Analysis

Initially, the data were double-entered into an Excel 2016 spreadsheet (Microsoft Corp), validated, and transported to SPSS software (version 27, IBM). Measures of central tendency and dispersion were used to describe quantitative variables, and absolute and relative frequencies were used to describe categorical variables. Subsequently, as it was a small sample with 2 independent groups from the same population, the Kolmogorov-Smirnov test was performed, concluding that there were large deviations from normality, and the *z* test was applied and analyzed at a significance level of 5%.

## Results

Among the 54 interviewees, there was a higher prevalence of women and the mean age of participants was 68.2 (SD 5.82) years. Most participants were married (n=25, 46%), retired (n=31, 57%), and had incomplete elementary education (n=28, 52%). Additionally, most had been treated for DM for 1 to 10 years (n=22, 41%) and self-reported a good quality of life (n=27, 50%; Table 1).

**Table 1:** Demographic and clinical profiles of older people with diabetes mellitus (DM) treated at the Hospital Universitário João de Barros Barreto geriatrics service between 2020 and 2022, Belem, Pará, Brazil (N=54).

Variable		Participants, n (%)
**Gender**		
	Women	38 (70)
	Men	16 (30)
**Age (years)**		
	60-64	22 (41)
	65-69	10 (19)
	70-74	13 (24)
	≥75	9 (17)
**Education**		
	Fundamental incomplete	28 (52)
	Elementary school complete	10 (19)
	High school complete	7 (13)
	Incomplete high school	6 (11)
	Illiterate	3 (6)
**Marital status**		
	Married or stable union	25 (46)
	Single	13 (24)
	Widower	9 (17)
	Divorced	7 (20)
**Occupation**		
	Retired or pensioner	31 (57)
	Self-employed	8 (15)
	Housewife	7 (13)
	Unemployed	5 (8)
	Other^a^	5 (8)
**Time of treatment for DM (years)**		
	1-10	22 (41)
	11-20	15 (28)
	21-30	9 (17)
	31-40	8 (15)
**Self-reported quality of life**		
	Good	27 (50)
	Regular	24 (41)
	Bad	3 (6)
**Discontinuation of hypoglycemic agents during treatment for COVID-19**		
	No	53 (98)
	Yes	1 (2)

^a^Other occupations included caregiver of older people and civil servant.

As for the clinical profile, it was observed that of the 54 interviewees, 48% (n=26) were infected by SARS-CoV-2. The most prevalent symptoms were fever followed by loss of taste and dyspnea. Among the 26 infected participants, 62% (n=16) were diagnosed with long COVID, presenting most commonly with fatigue or muscle weakness followed by myalgia. It is noteworthy that most of the respondents infected with COVID-19 did not discontinue their treatment for DM (Table 2).

**Table 2:** Clinical characteristics of older people with diabetes affected by COVID-19 and long COVID treated at the Hospital Universitário João de Barros Barreto geriatrics service between 2020 and 2022, Belem, Pará, Brazil.

Clinical characteristics			Participants, n (%)
**Symptoms of COVID-19 (n=26)**			
	Fever	17 (65)	
	Loss of taste	16 (62)	
	Dyspnea	15 (58)	
	Loss of sense of smell	14 (54)	
	Myalgia	10 (38)	
	Fatigue	3 (12)	
	Anorexia	2 (8)	
	Dry cough	5 (19)	
	Dizziness	2 (8)	
	Vomiting	2 (8)	
	Headache	1 (4)	
	Coryza	1 (4)	
**Symptoms of long COVID (n=16)**			
	Fatigue and/or muscle weakness	5 (31)	
	Myalgia	5 (31)	
	Alopecia	2 (13)	
	Persistent Fever	2 (13)	
	Anorexia	1 (6)	
	Dyspnea	1 (6)	

Table 3 shows the dimensions and items of the D-39 between waves. Overall, we noticed that the dimensions “sexual functioning” (mean 2.91, SD 2.11; *P*<.001) and “social overload” (mean 2.97, SD 1.76; *P*<.001) were unaffected, while the dimensions “energy and mobility” (mean 3.53, SD 1.71; *P*=*.*005), “diabetes control” (mean 3.60, SD 1.81; *P*<.001), and “anxiety and worry” (mean 3.96, SD 1.82; *P*<.001) had a higher score. Items 11, 20, 28, 31, 34, and 37 were not affected. Moreover, all other items were affected, especially items 14, 15, and 39. All results were statistically significant (Table 3).

**Table 3:** Dimensions and items of the Diabetes-39 instrument for older people with diabetes mellitus (DM) treated at the Hospital Universitário João de Barros Barreto geriatrics service between 2020 and 2022, Belem, Pará, Brazil (N=54).

Diabetes-39 instrument		Mean (SD)		*P* value		*z* score
**By dimensions**						
	Energy and mobility	3.53 (1.71)	.005		2.93	
	Diabetes control	3.60 (1.81)	<.001		2.94	
	Anxiety and worry	3.96 (1.82)	<.001		4.52	
	Social overload	2.97 (1.76)	<.001		2.36	
	Sexual functioning	2.91 (2.11)	<.001		2.38	
**By items**						
	1. Daily use of medication	3.44 (2.10)	<.001		3.38	
	2. Concern about financial issues	4.55 (1.82)	<.001		3.84	
	3. Decrease or lack of energy	3.88 (2.29)	.005		4.50	
	4. Follow prescribed treatment	3.62 (2.42)	<.001		2.96	
	5. Dietary restrictions	4.51 (2.24)	<.001		5.65	
	6. Worries about your future	4.44 (2.10)	<.001		3.50	
	7. Health problems other than DM	3.70 (2.33)	<.001		3.96	
	8. Stress or pressure in your life	3.66 (2.48)	<.001		3.53	
	9. Feeling of weakness	4.00 (2.40)	<.001		3.88	
	10. How far you can walk	3.44 (2.56)	<.001		3.34	
	11. Need for regular exercise	2.44 (2.24)	<.001		2.34	
	12. Loss or blurring of vision	3.77 (2.08)	<.001		4.46	
	13. Not being able to do what you want	4.62 (2.16)	<.001		3.65	
	14. Having diabetes	4.85 (2.36)	<.001		4.26	
	15. Losing control of sugar levels	4.81 (1.94)	<.001		4.07	
	16. Diseases other than diabetes	3.62 (2.35)	<.001		4.61	
	17. Having to test sugar levels	3.25 (2.37)	<.001		2.38	
	18. Time needed for control	3.37 (2.25)	<.001		3.65	
	19. Diabetes restrictions on family and friends	3.77 (2.29)	<.001		2.42	
	20. Embarrassment for having diabetes	2.85 (2.17)	<.001		2.00	
	21. Diabetes interfering with your sex life	3.22 (2.42)	<.001		1.11	
	22. Feeling of sadness or depression	3.22 (2.43)	<.001		2.61	
	23. Problems with sexual function	2.70 (2.31)	<.001		1.00	
	24. Try to keep diabetes under control	3.29 (2.23)	<.001		3.27	
	25. Complications due to your diabetes	4.62 (1.88)	<.001		4.03	
	26. Doing things that family and friends don’t do	3.33 (2.23)	<.001		2.11	
	27. Keep track of sugar levels	1.92 (1.81)	<.001		1.30	
	28. Need to eat at regular intervals	2.59 (1.88)	<.001		2.57	
	29. Not being able to do household activities	3.77 (2.54)	<.001		2.84	
	30. Decreased interest in sex	2.81 (2.11)	<.001		1.88	
	31. Having an organized routine for diabetes	3.00 (2.28)	<.001		2.57	
	32. Need to rest several times a day	3.25 (2.36)	<.001		4.11	
	33. Difficulties in climbing stairs	3.88 (2.50)	<.001		3.77	
	34. Difficulties in taking care of yourself	1.62 (1.71)	<.001		1.88	
	35. Agitated sleep	3.25 (2.28)	<.001		3.92	
	36. Walking slower than others	3.11 (2.30)	<.001		3.57	
	37. Being called diabetic	1.88 (1.76)	<.001		1.00	
	38. Having diabetes interfere in your family life	3.03 (2.27)	<.001		1.23	
	39. Diabetes in general	4.66 (2.03)	<.001		5.57	

## Discussion

Although research data on older people with diabetes is known worldwide, the assessment of their quality of life during pandemic times is still insufficient, and there is little research that has evaluated the effect of long COVID on the quality of life of this specific population. In this study, the sociodemographic profile showed a predominance of older women, corroborating other studies carried out in the Brazilian context [15-17]. The prevalence of older women with diabetes can be justified by the historical process of greater male mortality throughout life, which may be linked to lifestyle habits and health care, with this profile being observed worldwide [18].

Regarding age groups, there was a predominance of people between 60 and 64 years old, with a trend pointing toward longevity, approaching the Brazilian national estimate of 75 years old [19]. This result shows that older people have been seeking health services, mainly due to the severity of the pandemic in the country, and requiring specialized health care with the risks of COVID-19. However, we emphasize that in Brazil, the implementation of the Brazilian Ministry of Health’s National Policy for Comprehensive Men’s Health Care and access to health services for people aged 60 years or older is deficient [20,21].

The majority of older people interviewed had an incomplete education. This result can be explained by the difficulty in accessing school for women in the 20th century [22]. This fact corroborates the finding that women have little participation in work, contributing to the low level of education [23]. Studies have indicated that low education is correlated with lower quality of life scores [23,24].

When analyzing marital status, there was a predominance of married individuals and those in stable unions, corroborating a study carried out with older people with diabetes in northern Brazil [25]. A study carried out with older people showed that being married positively affected quality of life when associated with a couple, emphasizing that family members or other social support must be addressed as predictors of quality of life [26].

It was observed that the majority of older people were retired. Retirement predominates as the main source of income. Furthermore, it can be seen as a time for stress-free activities; it can be a time to find ways to start over, to take on projects, and to continue operating as a subject of one’s own destiny and as an agent in the family and society [25]. Another study pointed out that it may be a reason for attention, as this process is characterized by withdrawal from productive life and can lead to inactivity and sadness [26].

The treatment time for most elderly people for DM was relatively long, between 1 and 20 years. Previous reports have shown that the duration of treatment and adherence to drug therapy [27-29] coincide with the results of this study [27]. Regarding the quantity of medications used to control DM, there was a predominance of the use of few medications (ie, between 1 and 2). This differs from Brazilian studies that showed polypharmacy in individuals aged 60 years or older, which involved the use of a large number of medications [30]. These individuals are susceptible to the simultaneous involvement of dysfunctions in different organs or systems, and are therefore candidates for multiple use of medications.

It is interesting to note that despite the long period of DM, the majority of older people self-assessed their health as good. This result may be related to the fact that older people are cared for by a health service that offers specialized professional care, involving educational practices aimed at patients with diabetes [31,32].

Brazil is one of the countries most affected by the COVID-19 pandemic, with high estimates of morbidity and mortality from the disease a challenge shared by several countries. By October 17, 2022, the country had registered more than 4.6 million cases and 687,144 deaths from the disease. These numbers are growing at an unprecedented rate and pose significant challenges for the country and the health system due to the impact of losses, sequelae, and the future burden of disease [33].

In this sense, with regard to the clinical conditions of COVID-19, it is noteworthy that the majority of interviewees were infected by SARS-CoV-2, reflecting that the older population constitutes a vulnerable group and are at risk of infection by SARS-CoV-2, especially in those suffering from chronic diseases (eg, hypertension, diabetes, kidney disease, lung disease, and others), which increase the severity of the infection and its complications [7].

The WHO defines long COVID as a post–COVID-19 condition that occurs in individuals with a probable or confirmed history of SARS-CoV-2, generally 3 months after the onset of COVID-19, with symptoms that last at least 2 months and which cannot be explained by an alternative diagnosis [33]. In this study, the participants reported myalgia as the main symptom, followed by fatigue or muscle weakness, corroborating studies carried out in Korea [33] and the United Kingdom [34] with older people reporting myalgia and fatigue or muscle weakness as persistent symptoms.

The WHO points out that 10% to 20% of people who have recovered from SARS-CoV-2 suffer from post–COVID-19 symptoms, which can last more than a year [35]. In this regard, the WHO recommends that governments implement integrated care for patients suffering from this condition, including rehabilitation for the treatment of long COVID [34,36]. At the research site of this study, an outpatient rehabilitation clinic for long COVID was created, and patients are monitored by health professionals, mainly with assistance from physiotherapists.

In this study, when analyzing the distribution of responses from interviewees related to the “social overload” dimension, we found that it was not affected. These results corroborate a previous study [37] on Latin American culture that highlighted the priority of help from friends and family and are probably related to the psychosocial coping of older people with diabetes. The period of the COVID-19 pandemic appears to have alleviated the social burden of older people with diabetes [38].

Regarding the “sexual functioning” dimension, we found that it was also not affected. This result differs from studies carried out with older people in Brazil [38] and Chile [39], which showed that older people with diabetes were negatively impacted during the pandemic. This result deserves attention in educational programs on diabetes that address sexual life, as it is a good strategy to facilitate the approach to aspects related to DM in sexual life [36]. Furthermore, a study carried out with older people showed that attention and treatment for sexual problems improves quality of life [37].

On the other hand, the “anxiety and worry” dimension was the most affected. This result corroborates international studies carried out in Nepal [40] and Thailand [41]. In Brazil, a study [38] carried out with older people reported worsening in this dimension, as evidenced by the loss of vitality and mobility.

The items “having diabetes” and “diabetes in general” were the most negatively impacted, which may be related to low education [36] and is similar to a result found in Mexico [42]. In this sense, it is necessary to draw up a care plan by the multidisciplinary team that provides care to older people with diabetes with the aims of demystifying the diagnosis of DM and informing them about the importance of family integration in the new lifestyle with a view to achieve an improvement in their quality of life.

### Limitations

The study has limitations due to the sample size and the evaluation of patients being restricted to a single hospital outpatient clinic during a pandemic period with restrictions on appointments. However, the results obtained in this research contribute to comprehending the quality of life of older people with diabetes and long COVID, thus allowing knowledge of possible actions to be implemented in the health care of this specific population.

We suggest carrying out other studies with larger samples to clarify the impact of long COVID on the quality of life of older people with diabetes. Longitudinal studies would be important to investigate whether quality of life can change over time or in relation to older people with diabetes suffering from long COVID.

Thus, this study supports the new perspective of care for older patients with DM and long COVID. In this sense, comprehensive care and monitoring of older people are an important strategy given that there is an increasing need to work on quality of life, whether due to the aging of the population or the reduction of the sequelae imposed by SARS-CoV-2.

### Conclusions

We conclude that the quality of life of older people with diabetes in the dimensions of “social overload” and “sexual functioning” proved to be little affected or not affected at all, while the opposite was true for the other dimensions, more specifically “anxiety and worry.” Among those affected, we observed long COVID as a recent peculiarity in the aging process. In this sense, we suggest the implementation of public policies capable of being carried out in this new reality in the postpandemic period. Future research should be developed on this theme, since understanding the quality of life of older people with diabetes in a pandemic context contributes to subsidizing health actions aimed at improving the quality of life of this specific population.

## Abbreviations

D-39: Diabetes-39
DM: diabetes mellitus
STROBE: Strengthening the Reporting of Observational Studies in Epidemiology
WHO: World Health Organization

## Data Availability

The data sets used or analyzed during this study are available and under the domain of the corresponding author upon plausible justification or referred to in the informed consent.
